# How to Start Interventional Radiology

**DOI:** 10.5812/ircmj.16619

**Published:** 2013-12-05

**Authors:** Hossein Ghanaati, Kavous Firouznia, Amir Hossein Jalali, Madjid Shakiba

**Affiliations:** 1 Department of Radiology, Advanced Diagnostic and Interventional Radiology Research Center (ADIR), Tehran University of Medical Sciences, Tehran, IR Iran

**Keywords:** Interventional Radiology, Research, Procedure, Embolization

## Abstract

Interventional techniques aim to find safer and better ways to treat vascular diseases even in many instances, the interventional radiology solutions has been considered the only treatment option for the patients. Interventional radiologists are specialists who perform minimally invasive procedures instead of surgery or other treatments. These procedures apply various imaging and catheterization procedures in order to diagnose and treat diseases. In each country, interventional radiology practice establishment of varies according to local factors, but following a standard strategy seems better to set up this facility. According to above mentioned points, we decided to establish this specialty in our hospital since 2001 as the pioneer center in Iran. In this presentation we will discuss about our experience for start interventional radiology.

## 1. Introduction

Interventional radiology is a subspecialty of radiology in which minimally invasive procedures are done using image guidance. Interventional techniques, such as injecting arteries with dye, visualizing these via x-ray, and opening up blockages, developed from early pioneers' bold and sometimes controversial experiments which aimed to find safer and better ways to treat vascular disease (1-4). The benefits of interventional techniques thus are reduced recovery time and pain associated with the procedures, due to their less invasive nature (1, 5-8). In each country, interventional radiology practice varies according to local factors. Furthermore, in some countries, interventional radiology is formally recognized as a unique subspecialty of diagnostic radiology, whereas in other countries interventional radiology is formally recognized as a distinct radiologic specialty. We established interventional radiology in Iran since 2001 starting our experience with osteoid osteoma photocoagulation therapy, laser therapy for the lower extremities varices, PLDD, and PRT (9-11). Consequently, we accomplished development by partnership with other clinicians and being recognized by primary care responsibilities and the public. Vascular interventions especially fibroid embolization, chemoembolization, aneurysm coiling, brain AVM embolization have been other achieved milestones (12-19).

Now we are noticed for dedicated and standardized training programs for professionals together with performing research and developing our cooperation with pioneer countries. For example we have short term courses in interventional radiology. In the future we will continue our forward movement towards developing new techniques, devices, and broadening interventional treatment indications. Finally, development of subspecialties such as neurointervention in the field of interventional radiology is another objective that has been planned.

## 2. Establishing Instruments and Specialists

We started to have a dedicated imaging center in Imam Khomeini university affiliated hospital since 1995.

By inviting famous interventionalists in different fields from developed countries we started to learn more and also some of our staff has been participated in short practical intervention courses.

This center is unique in our country.

Imam Khomeini Hospital has more than 2000 beds and different institutes, and we have one of the most modern centers in the Middle East.

We have a lot of equipments such as 3 tesla MRI, 1.5 tesla MRI, 64 slice MDCT scan, three angiography units (two of them are active and last one will be active soon).

There are different clinics for example we have low back pain clinic to manage low back pain by interventional procedures and during these 16 years we have treated more than 4000 patients who had low back pain (10).

We are going to modify some techniques according to regional conditions to make some interventional techniques more economic.

Our oncointervention team is very skillful and we do RFA, TACE, portal vein embolization and cementoplasty. Also we do interventional pain management for cancer patients (15-19).

Oncointervention team now is important member of tumor board of hospital.

We have a lot of HCC unfortunately in Iran and we treatment them under chemoembolization (15-19).

We have Research projects on uterine artery embolization. One of them is pregnancy after embolization and found answer to this question. We did to now more than 1000 cases of uterine artery embolization. We have a lot of citation to our papers about UAE (12-14).

And we have a lot of vascular cases including aortic stenting ,infra popliteal angioplasty and etc. because of high road accident rate in Iran we have a lot of cc fistula and we have more than 200 cases we managed them by coiling or by putting balloon to close fistula (20, 21).

For the aneurysm we do coiling; stent assistant coiling; balloon assistant coiling and flow diversion technique.

This center is referral for neurointervention and we embolized more than 700 brian AVMs. 

Recently we make dedicated stroke center and neurovascular department with modern ICU beds and biplane angiography unit.

Research Center of Advanced Diagnostic and Interventional Radiology (ADIR)

Advanced Diagnostic and Interventional Radiology Research Center was founded in Medical Imaging Center of Tehran University of Medical Sciences in summer 2009. Given essential attention to the position of the Medical Imaging Center which is located in one of the largest referral academic centers in Iran affiliated to Tehran university of medical sciences; existence of advanced facilities such as multi-detector 64 slice CT scan¸ MRI 3tesla¸ flat panel angiography¸ advanced ultrasonography devices and other facilities; and the presence of high-skilled, experienced and qualified specialists we determined to establish the this center which is pioneer in the field of radiology in Iran.

Our goals for developing research center are:

1) Development and application of human knowledge on radiology

2) Performing basic, clinical and epidemiologic researches for improving the health system

3) Collection, regulation, and classification of documents and articles, and publication of them

4) Training researchers in the field of radiological sciences

5) Motivation, persuasion, and employment of researchers

6) Drawing the attention and use the help of related research centers in the country

7) Scientific cooperation with research and training centers in other countries and international organizations

## 3. International Collaboration

The start of cooperate with advance interventional center out of country is one of the projects that we did for the first time on human with supervision of San- Diego university (22, 23).

And we have a lot of patients coming from neighbor country to Iran to receive medical services; some of them because lack of service and some of them come to Iran due to the more economical services.

Turkey is very advanced in medicine but some people come to Iran to take services in Tehran or other cities because of the price.

## 4. Educational Activities

Also we have cooperation with all institutes in Tehran such as a project that we have participated in transplant islet cell to treat typically diabetic patients (24). Also we have some project in stem cell (25-27). Iran is one of the countries for pilot of stem cell management and that we help patients to inject stem cell into the hepatic artery to treatment decompensate cirrhotic patients.

I travel to all cities in my country to help Colleagues to do common work and also a lot of my Colleague come to Tehran learn more about intervention. Now we have more formal programs on intervention and we have the first fellowship course that started in IRAN and graduated specialists are working after fellowship. We have cooperation with industry to make medical instruments.

In conclusion this rapid growth in 17 years related to innovative hard working persons, the good policy of ministry of health, availability of modern instruments, and help of known professors from USA, Europe and other advanced countries. We are ready to exchange our experience and cooperate with our Colleagues in the world.
